# Advancing the clinical science of creativity

**DOI:** 10.3389/fpsyg.2014.00613

**Published:** 2014-06-19

**Authors:** Marie J. C. Forgeard, Jeanette G. Elstein

**Affiliations:** Department of Psychology, Positive Psychology Center, University of PennsylvaniaPhiladelphia, PA, USA

**Keywords:** creativity, psychopathology, clinical psychology, psychotherapy, flexibility

Can the therapeutic benefits of creativity explain its documented association with psychopathology (Andreasen, 1987; Ludwig, 1995)? Past research seems to have devoted most of its attention to another hypothesis in order to explain this relationship: that features of some disorders may be beneficial for creative cognition (especially in the arts)—for example, the racing thoughts, energy, and openness characteristic of hypomania in bipolar disorder (Johnson et al., 2012), or the rumination observed in depression (Verhaeghen et al., 2005). Other explanations, however, should not be ignored or considered mutually exclusive. Creative work may sometimes exacerbate psychopathology. For example, Kaufman and Baer (2002) suggested that poets may be especially susceptible to mental illness because poetry requires emotional expression and introspection, and unlike prose, may not provide adequate opportunities for making meaning out of one's experience. Conversely, and leaving aside third variable explanations (which also deserve further research), we explore the hypothesis that psychopathology may motivate individuals to engage in creative activities as a way to alleviate their suffering and enhance their well-being. To date, two main empirical literatures have examined this claim. First, reviews of art therapy trials have found that such interventions typically lead to small but statistically significant improvements on a range of psychological measures (Slayton et al., 2010; Forgeard and Eichner, 2014; Maujean et al., 2014). Second, studies examining the benefits of “everyday creativity” suggest that engaging in day-to-day creative activities may both reflect and foster psychological health (Richards, 2007). In keeping with this, findings of a recent experience-sampling study showed that young adult participants were more likely to be engaged in creative activities than other activities when they reported feeling happy and active (Silvia et al., 2014).

In spite of these efforts, important gaps exist in our understanding of the therapeutic benefits of creativity. The first and foremost of these gaps is the following: *to the best of our knowledge, little empirical evidence has demonstrated that creative thinking per se is one of the specific active ingredients accounting for the benefits of creative activities.* To date, past research has investigated the role of other potential mechanisms including adaptive emotion regulation, flow, meaning-making, or growth from adversity in order to explain the benefits of creative activities (Csikszentmihalyi, 1996; Drake and Winner, 2012; Forgeard et al., 2014). Thus, it remains unclear whether the benefits of creative activities are due to creative thinking, or to other factors. We propose that the time is ripe to collect such evidence in order to provide a richer understanding of the nature of the therapeutic benefits of creative thinking. We outline a research agenda to advance the clinical science of creativity from a cognitive-behavioral perspective.

## Creative thinking as a transdiagnostic process

Clinical scientists are developing a growing interest in understanding transdiagnostic processes (i.e., processes shared across disorders) that can account for overlap in symptoms and high rates of comorbidity between psychological disorders, as well as recovery or resilience. These processes (whether pathological or adaptive) can help develop parsimonious theories of disorder and health, as well as pragmatic treatments (Mansell et al., 2009; Forgeard et al., 2011). The research agenda we present here is based on the following hypothesis: Creative thinking constitutes an important yet understudied transdiagnostic process that can be defined, operationalized, assessed, and (if found to be adaptive) enhanced. Creativity refers to *the generation of ideas or products that are both novel (i.e., original, unusual) and useful (i.e., valuable, helpful)* (Stein, 1953; Runco and Jaeger, 2012). Creative thinking can be subjective (i.e., novel and useful to the self) and/or in comparison to others (i.e., novel and useful to all) (Kaufman and Beghetto, 2009). It is also not reserved to prototypical creative domains (e.g., the arts and the sciences), but is present to varying degrees in almost all areas of life—excelling at work, solving thorny interpersonal problems, managing painful emotions, or cooking dinner, are all tasks that may benefit from effective creative thinking. Related to this, creative thinking takes place not only in “creative therapies” (e.g., art therapy), but to some degree also in all forms of psychotherapy.

How does creativity relate to other processes already studied by clinical scientists? Creative thinking is by definition closely related to prospection, defined as the mental representation of possible futures (Seligman et al., 2013). Past research suggests that maladaptive patterns in future-oriented thinking play a key role in psychopathology (Miloyan et al., 2013). For example, both anxious and depressed individuals tend to overestimate future negative outcomes, and depressed individuals also tend to underestimate future positive outcomes (e.g., MacLeod and Byrne, 1996; Miranda and Mennin, 2007). How might generating novel and useful ideas influence the extent to which individuals think about and prepare for the future in a constructive manner? Creativity may contribute to adaptive prospection by enhancing another closely related process: psychological flexibility, defined as the ability to effectively adapt one's cognitions, emotions, and behaviors to the situation at hand (Kashdan and Rottenberg, 2010). Psychological flexibility does not necessarily require creative thinking—individuals may build a repertoire of options by learning from others or from the environment (as opposed to inventing them anew). We propose, however, that creative thinking probably enhances and strengthens psychological flexibility by allowing individuals to generate new and effective cognitive, emotional, and behavioral strategies on their own. Creative thinking may therefore help counteract a number of detrimental transdiagnostic processes reflecting maladaptive prospection and inflexibility, including repetitive negative thinking, as well as interpretational and expectancy biases (Harvey et al., 2004) by helping individuals adopt adaptive interpretations and coping styles (Fresco et al., 2006).

## Examining creative thinking as an active ingredient

What comes next for clinical scientists interested in examining whether and how creative thinking promotes flexibility and decreases psychopathology? Treatment outcome researchers should continue to build empirical support for the efficacy of interventions thought to rely on creative thinking (e.g., art therapy) (Kaplan, 2000; Gilroy, 2006; Maujean et al., 2014). Randomized controlled trials (RCTs) remain the gold standard for this purpose and are necessary to establish that an intervention is empirically supported, among other criteria (Chambless and Hollon, 1998). Of course, preliminary investigations such as single case designs, or uncontrolled trials, often provide useful insights.

Aside from outcome research, rigorous process research is needed in order to test whether creative thinking itself (as opposed, or in addition to, other mechanisms) is one of the active ingredients accounting for positive outcomes. Process research uses appropriate research designs and mediation analyses in order to test causal mechanisms responsible for the effects of an intervention (Kazdin, 2007). In addition to assessing the contribution of creative thinking to outcomes, researchers should also further assess the mediating role of mechanisms examined in prior scholarship (including adaptive emotion regulation, flow, meaning-making, or growth from adversity, as mentioned above), as well as additional mechanisms such as psychological flexibility (Kashdan and Rottenberg, 2010), behavioral activation (Jacobson et al., 2001), or self-efficacy (Bandura, 1997), among others.

Future research should examine the extent to which creative thinking *per se* contributes to these processes, and in turn, to psychological adjustment. Such research is needed to establish whether creative thinking holds special benefits for well-being compared to other thinking styles. This assessment in no way diminishes the value of previous findings, but rather highlights the importance and value of addressing this question in future research. Similarly, little research has investigated whether and how creative thinking abilities contribute to the effects of other forms of therapy. For example, cognitive therapy for depression encourages individuals to generate alternative explanations for automatic thoughts and to assess cognitions for accuracy and usefulness (Beck et al., 1979)—a process which could recruit and/or develop creative thinking abilities.

It is not just on the client's end that creative thinking may enhance outcomes—therapists too need to be creative thinkers. Concerns have been raised about the extent to which manualized treatments can help clients whose symptoms are more complex than those included in RCTs (Westen et al., 2004). Yet, although manuals are required to operationalize and demonstrate the efficacy of a treatment, most researchers and clinicians tend to agree that good manuals leave space for “flexibility within fidelity” in order to effectively tailor treatment to clients' specific concerns and learning styles (Kendall and Beidas, 2007). Therapists' creative thinking abilities therefore probably enable them to flexibly invent new ways to faithfully implement treatments (Deacon, 2000). Within the context of cognitive-behavioral therapy, such creative thinking may be manifested in astute behavioral experiments to test negative cognitions, individualized exposures for anxiety disorders, or compelling metaphors to foster motivation and change (Peterman et al., in press).

## Conclusion

Researchers interested in advancing the clinical science of creativity have exciting tasks ahead of them: to continue building empirical support for the value of creative therapies using outcome research, and to investigate the role of creative thinking as a transdiagnostic process that may promote adaptive future-thinking and psychological flexibility using process research. These endeavors will enrich our understanding of the relationship between creativity, psychopathology, and health by investigating the circumstances under which creative thinking is or is not beneficial, and by identifying the metacognitive strategies that help individuals tell the difference (Kaufman and Beghetto, 2013). In particular, it is likely that original thinking may only be beneficial in moderate amounts or in certain situations, though more research is needed to test this claim. Related to this, researchers have called for investigating the boundary conditions under which any positive psychological trait or process may become detrimental, as seemingly linear relationships may in fact be nonmonotonic when examining their full range of expression (Grant and Schwartz, 2011). The optimal “dose” of originality and flexibility may therefore vary according to the situation at hand. For example, a person might benefit from considering a wide array of options in order to repair a romantic relationship after a fight. A simple “I am sorry” may not be as effective as an apology expressed in a clever and constructive way. Past a certain point however, the search for novel and flexible solutions may lead to impulsivity or instability (Kashdan and Rottenberg, 2010). In this case, organizing a last-minute unusual and extravagant date or writing an entire book of poems to apologize could be perceived as “too much of a good thing.”

In addition, future research should further examine how various forms of creativity relate to well-being, given that past research in this area has mainly explored the effects of artistic creativity. For example, past research suggests that artists suffer from psychopathology at a greater rate than scientists (Ludwig, 1995). These findings could be influenced by self-selection effects, and/or by the possibility that the creative process has differential benefits for artists vs. scientists. Related to this, the extent to which creative thinking benefits well-being may depend on whether the creative work at hand focuses on one's personal situation or mental state (a case perhaps more typical of the arts) or on an external problem (a case perhaps more typical of the sciences). Thus, future research should further investigate whether and how creative work affects well-being in fields other than the arts.

In light of past research in this area, as well as the promise of addressing existing remaining questions highlighted here, we believe that the study of the therapeutic benefits of creativity will continue to make important contributions to clinical science by further investigating one of the possible causal mechanisms accounting for the relationship between creativity, psychopathology, recovery, and resilience.

### Conflict of interest statement

The authors declare that the research was conducted in the absence of any commercial or financial relationships that could be construed as a potential conflict of interest.
